# Isolation, Characterization, Antioxidant and Anticancer Activities of Compounds from *Erythrina caffra* Stem Bark Extract

**DOI:** 10.3390/antiox14091035

**Published:** 2025-08-22

**Authors:** Femi Olawale, Olusola Bodede, Mario Ariatti, Moganavelli Singh

**Affiliations:** 1 Nano-Gene and Drug Delivery Group, Discipline of Biochemistry, University of KwaZulu-Natal, Private Bag X54001, Durban 4000, South Africa; olawalefemi3@gmail.com (F.O.); ariattim@ukzn.ac.za (M.A.); 2 Biodiscovery Centre, Department of Chemistry, University of Pretoria, Pretoria 0028, South Africa; olusolabodede@northeastern.edu

**Keywords:** *Erythrina caffra*, anticancer, antioxidant, bioactivity, phytochemicals, ferulate, apoptosis

## Abstract

*Erythrina caffra* is a traditional plant used to treat cancer and inflammation. The study aimed to assess and isolate anticancer compounds from *E. caffra* bark. The plant material was extracted sequentially in n-hexane, dichloromethane, ethyl acetate and methanol. The 2,2-diphenyl-1-picrylhydrazyl (DPPH) radical scavenging and 3-(4,5-di methyl thiazol-2-yl)-2,5-diphenyltetrazolium bromide (MTT) assays were used to evaluate the crude extracts’ antioxidant and anticancer activities, respectively. Column chromatography was used to purify the potent extracts of the stem bark in order to isolate the bioactive compounds. The crude extracts of the *E. caffra* bark demonstrated antioxidant and anticancer activity, with the dichloromethane (DCM) extract producing the most favorable activity. Three compounds, namely Hexacosanyl isoferulate, Tetradecyl isoferulate, and 1-Heneicosanol, were detected in fractions from the DCM extract. All the isolated compounds showed significant anticancer potential, with the hydroxycinnamic acid compounds showing better anticancer effects in the cervical (HeLa) and breast cancer (MCF-7) cells. The compounds showed greater activity than even the standard drug, 5-fluorouracil, in the MCF-7 cells, with the tetradecyl isoferulate and hexacosanyl isoferulate fractions having IC_50_ values of 123.62 and 58.84 µg/mL, respectively. The compounds were observed to be capable of triggering caspase cascade events, leading to apoptotic cell death. Overall, *E. caffra* extracts contained important bioactive compounds that induced apoptotic cell death in HeLa and MCF-7 tumor cells, warranting further investigations in vitro and in vivo.

## 1. Introduction

Since the mid-20th century, there has been a surge in the incidence of cancer. This situation has been linked to a variety of unhealthy changes, such as lifestyle, diet, environment, and diseases [1]. According to GLOBOCAN estimates for 2022, about one in five individuals, both men and women, will be diagnosed with cancer during their lifetime, while approximately one in nine men and one in twelve women will die from the disease [2]. The problem of cancer is far from solved, despite the many successful treatments that have been developed over the years. As the incidence of cancer continues to rise, finding effective and safe treatments for this disease remains a priority. Among the available cancer treatments, chemotherapy is still one of the most widely used and successful approaches. Chemotherapy has proved to be a useful procedure in the fight against cancer since it helps to reduce tumor size and prevent cancer from spreading to other parts of the body [3]. Despite the advances made in chemotherapy, the search for improved treatments and better outcomes for cancer patients continues. The use of medicinal plants as a source of biologically active compounds, including antioxidants and anticancer drugs, is a promising area of research. Many plant-derived compounds, such as paclitaxel and vincristine, have already made significant contributions to cancer therapy [4]. With the increasing success of this approach, wider research is needed to develop more effective treatments and to better understand the potential of plants in disease management.

One of the interesting medicinal plants with therapeutic potential is *Erythrina caffra (E. caffra*. Commonly known as the coastal coral tree, it is an ethnomedicinal plant belonging to the family Fabaceae. Traditional healers in Southern Africa have long utilized various parts of the plants for the treatment of cancer, inflammation and wounds [5]. *E. caffra* contains diverse bioactive compounds such as alkaloids, flavonoids, terpenoids, phenolics and saponins [6]. The extracts from *E. caffra* have been found to have a number of biological and pharmacological activities. They have been shown to possess antioxidant, anti-inflammatory, and antimicrobial properties, linked to the presence of several bioactive constituents in the plant [7].

In recent years, a number of experimental studies have been conducted to examine the biomedical properties of extracts from *E. caffra* [8,9]. The findings of these studies have been promising, indicating that *E. caffra* extracts could be a potential source of natural therapeutic and anticancer agents [8,9]. Moreover, some researchers were able to identify several phytoalexins from the root bark of *E. caffra*, including caffraisoflavan I and caffraisoflavan II, erystagallin A, erythrabyssin I, erythrabyssin II, abyssinone II, abyssinone IV and abyssinone V [9]. These studies point to the therapeutic and anticancer prospects of *E. caffra*. Despite the significance of these studies, there are currently no reports on bioactive anticancer compounds from the stem bark of *E. caffra*. In addition, the underlying mechanism implicated in the anticancer effects of *E. caffra* remains unclear. Considering that phytochemical profiles can vary based on geographical origin, plant part, and environmental conditions, further investigation is warranted [10].

Taking these conditions into account, the current study aims to evaluate the anticancer potential of *E. caffra* from South Africa and purify bioactive anticancer compounds that could be developed as drug candidates for cancer treatment.

## 2. Materials and Methods

### 2.1. Materials

Hexane (>99%, CAS 110-54-3), dichloromethane (>99%, CAS 75-09-2), ethyl acetate (>99%, CAS 141-78-6), methanol (>99%, CAS 67-56-1), ascorbic acid, phosphate-buffered saline (PBS) tablets, sodium carbonate, dimethyl sulfoxide (DMSO), and 3-(4,5-dimethylthiazol-2-yl)-2,5-diphenyl tetrazolium bromide (MTT) were procured from Merck (Darmstadt, Germany). The 2,2-diphenyl-1-picrylhydrazyl (DPPH), acridine orange, and ethidium bromide were obtained from Sigma Aldrich (St Louis, MO, USA). Cell culture reagents: Eagle’s minimum essential medium (EMEM), fetal bovine serum (FBS), trypsin and antibiotics (Penicillin (5000 units/mL)/Streptomycin (5000 µg/mL)) were purchased from Lonza Bio-Whittaker (Verviers, Belgium). All sterile tissue culture plasticware was supplied by Corning Inc. (New York, NY, USA). The human embryonic kidney (HEK293, CRL-1573), cervical cancer (HeLa, CCL-2) and breast cancer (MCF-7, HTB-22) cells were originally sourced from the American Type Culture Collection (Manassas, VA, USA), with mycoplasma testing performed routinely before in vitro studies. All other reagents were of analytical grade and were locally sourced. The experiments were conducted as presented in the workflow in Supplementary Figure S1.

### 2.2. Plant Collection

The *E. caffra* stem bark was collected from a tree growing within the University of KwaZulu-Natal, Westville (29.8674° S, 30.9807° E). The plant was authenticated at the Department of Biology, University of KwaZulu-Natal, and a voucher specimen (F. Olawale 1) was deposited in the Ward Herbarium of the university.

### 2.3. Sample Extraction

The harvested bark was air-dried and pulverized. Subsequently, about 1 kg of the sample was subjected to sequential solvent extraction in n-hexane, dichloromethane, ethyl acetate and methanol by continuous stirring in a mechanical shaker at 80 rpm for 72 h. The filtrate was concentrated using a rotary evaporator, air-dried to a constant weight and the crude extracts obtained were analyzed for their antioxidant and anticancer activities.

### 2.4. Antioxidant Study by 2,2-Diphenyl-1-Picrylhydrazyl (DPPH) Assay

The antioxidant potential of the crude extracts was evaluated by measuring their DPPH radical scavenging activity following the protocol described by Bukhari et al. [11]. Approximately 50 µL of a 0.3 mM DPPH solution (in methanol) was incubated with 100 µL of the extracts or ascorbic acid standard (with varying concentrations from 50 to 250 µg/mL) in 96-well plates. A control well containing 5% dimethyl sulfoxide (DMSO) and DPPH was run concurrently. The samples were incubated in the dark for 30 min at room temperature. The absorbance was read against a DMSO blank at a wavelength of 517 nm using a microplate reader. DPPH scavenging activity was evaluated using Equation (1).

(1) $$\mathrm{DPPH}\%\;\mathrm{radical}\;\mathrm{scavenging}\;\mathrm{activity}=\frac{Ac-As}{Ac}\times 100$$

where Ac = absorbance of control, and As = sample absorbance

The IC_50_ values were calculated directly using GraphPad Prism version 9.0, where the data were used to fit a linear or non-linear regression model.

### 2.5. MTT Cytotoxicity Assay

Cytotoxicity was evaluated using the MTT (3-(4,5-dimethylthiazol-2-yl)-2,5-diphenyltetrazolium bromide) assay to measure cell viability after treatment with the plant extracts. This assay measures the percentage viability of a cell population by monitoring the ability of the NAD(P)H-dependent oxidoreductase enzyme system in live cells to reduce MTT to formazan, an insoluble purple form of the dye [12]. Confluent MCF-7 cells were seeded in 96-well plates at 2.0 × 10^4^ cells/well and incubated for 24 h at 37 °C in 5% CO_2_. The medium was replaced with fresh medium, and the extracts were added in varying concentrations (50–250 µg/mL). The plates were incubated at 37 °C in 5% CO_2_ for 48 h. At the end of the incubation period, the spent medium was discarded and replaced with 100 µL fresh medium containing 10 µL (5 mg/mL in PBS) MTT reagent. The cells were then incubated for an additional 4 h. The MTT/medium was subsequently removed, and 100 μL DMSO was added to solubilize the formazan dye product. Absorbance at 570 nm was assessed using a microplate reader (Vacutec, Hamburg, Germany). Cell viability was calculated as in Equation (2).

(2) $$\mathrm{Cell}\;\mathrm{viability}\;(\%)=\frac{At}{Ac}\times 100$$

where At = absorbance of test or treated cells and Ac = absorbance of control or untreated cells.

### 2.6. Acridine Orange/Ethidium Bromide Dual Staining for Apoptosis

A qualitative assessment of apoptosis induction in cancer cells was conducted by the acridine orange-ethidium bromide (AO/EB) dual staining method, as described previously [13,14]. Confluent cells (4.0 × 10^3^ cells/well) were trypsinized and seeded into 48-well plates and incubated for 24 h at 37 °C in 5% CO_2_. Subsequently, the spent medium was replaced, and cells were incubated for a further 48 h with the extracts or standard drug (5-fluorouracil) at a concentration of 250 µg/mL. The cells were rinsed twice with cold PBS, and 15 µL of the AO/EB dye mixture (0.1 mg/mL: 0.1 mg/mL) was added, and the cells were incubated for 5 min at ambient temperature. The cells were washed with PBS to remove excess dye and then visualized under an Olympus fluorescence microscope (Wirsam Scientific and Precision Equipment Ltd., Johannesburg, South Africa). Images were captured using a CC12 fluorescence camera and Analysis Five Software (Olympus Soft Imaging Solutions, Olympus, Tokyo, Japan) at 200× magnification.

### 2.7. Isolation of Bioactive Fractions of E. caffra and NMR Characterization

Column chromatography (CC) was conducted by the wet packing method using silica gel 60 (0.040–0.063 mm, Merck, Darmstadt, Germany) and n-hexane. A 4 g portion of the crude dichloromethane (DCM) extract was subjected to column chromatography using hexane/DCM as the mobile phase. The column was eluted using a gradient solvent system starting with a mixture of 100% n-hexane, n-hexane/DCM, and 100% DCM. From the column chromatography, a total of 19 aliquots (about 100 mL) were collected, which were later spotted on thin-layer chromatography (TLC) plates (silica gel 60 F_254_). The plates were first subjected to UV irradiation at 254 nm and 366 nm. This was followed by spraying with 10% sulfuric acid in methanol (MeOH) solution, and heating to visualize the fractions on the plates. Similar aliquots were combined into four fractions: fraction 1 (1–8), fraction 2 (9–13), fraction 3 (14–16), and fraction 4 (17–19).

### 2.8. Qualitative Analysis

Fraction 1 yielded compound 3, fraction 3 yielded compound 2, and fraction 4 yielded compound 1, as determined by the ^1^H and ^13^C NMR and HRMS analysis.

#### 2.8.1. NMR Characterization

The fractions were later analyzed via NMR spectroscopy and mass spectrometry. ^1^H and ^13^C NMR spectra were recorded in deuterated chloroform (CDCl_3_) at room temperature on a Bruker Avance III 400 MHz spectrometer (Bruker Corporation, Johannesburg, South Africa).

#### 2.8.2. GC–MS Characterization

A single quadrupole Shimadzu GC–MS GC–MS-QP2010SE gas chromatograph–mass spectrometer (Shimadzu, Shiga, Japan) was used to analyse the phytochemicals from the plant extract, by direct injection of the sample. The data interpretation and peak identification were carried out by comparing the molecular spectra to those of substances with related spectra in the National Institute of Standards and Technology (NIST) database.

#### 2.8.3. High-Resolution Mass Spectrometry (HRMS)

High-resolution electrospray ionisation mass spectra of the samples were obtained on a Waters Micromass LCT Premier TOF-MS instrument (Waters Corporation, Milford, MA, USA).

### 2.9. MultiCaspase Analysis

The multicaspase assay was performed using the Muse MultiCaspase kit, which can detect the presence of multiple caspases (caspase-1, 3, 4, 5, 6, 7, 8, and 9). The kit utilizes a derivatized VAD peptide that can detect the activity of multiple caspases and a dead cell dye that provides information on membrane integrity or cell death. Cells were seeded into 48-well plates at a density of 1.0 × 10^5^ cells/well and allowed to attach over 24 h. Subsequently, 100 µg/mL of the isolated fractions were added, and the cells were incubated for 48 h. The cells were then trypsinized and reconstituted in 1 × caspase buffer. Approximately 5 μL of the Muse™ MultiCaspase working solution was added to 50 μL of cell suspension and incubated at 37 °C for 30 min. Thereafter, 150 μL of 7-AAD working solution was added, mixed thoroughly, and analyzed using the Muse™ cell analyzer (Luminex Corporation, Austin, TX, USA).

### 2.10. Statistical Analysis

The data in this study are presented as mean ± standard deviation (SD) from three independent replicates (*n* = 3). Statistical analysis was performed using GraphPad Prism version 9.0 (GraphPad Software Inc., San Diego, CA, USA). One-way analysis of variance (ANOVA) was used to assess statistical differences, and Tukey’s post hoc test was applied when the ANOVA results were statistically significant (*p* < 0.05).

## 3. Results

### 3.1. Antioxidant and Anticancer Activity of Crude Extracts of Erythrina caffra Stem Bark

**Figure 1:**
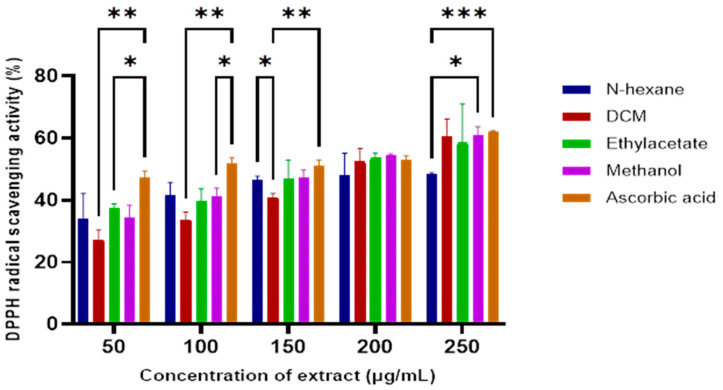
The DPPH radical scavenging activity of *E. caffra* stem bark extracts compared to the ascorbic acid standard. Data are presented as mean ±SD (*n* = 3). * *p* < 0.05, ** *p* ≤ 0.01, *** *p* ≤ 0.001, for statistical significance.

Crude extracts from the sequential extraction of the *E. caffra* stem bark were evaluated for antioxidant activity using the DPPH radical scavenging assay. As illustrated in Figure 1, increasing concentrations of the extracts corresponded to increased DPPH scavenging activity. The dichloromethane (DCM) extract exhibited the most potent antioxidant activity from all tested extracts, with an IC_50_ value of 144.17 µg/mL (Table 1). The antioxidant activity index of these extracts was comparable to that of ascorbic acid, indicating their potential in chemoprevention.

Although no prior reports exist on the antioxidant activity of the *E. caffra* bark specifically, earlier studies of the Erythrina genus have shown significant antioxidant capacity both in vitro and in vivo [7,15,16]. Since oxidative stress can cause DNA damage, leading to altered signaling and cancer development, the antioxidant properties of the extracts could contribute to cancer prevention.

**Figure 2:**
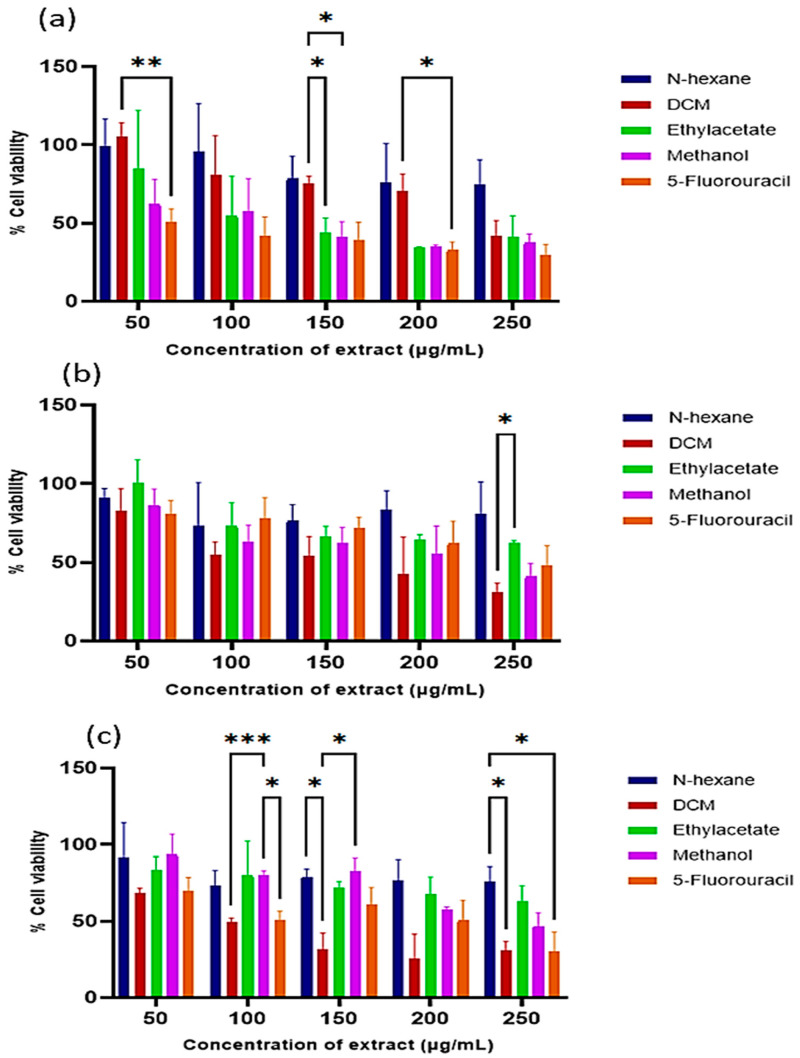
Cytotoxicity of the *E. caffra* bark extracts on: (**a**) HEK293, (**b**) HeLa, and (**c**) MCF-7 cells. Data are presented as mean percentage cell viability ± SD (*n* = 3). * *p* < 0.05, ** *p* ≤ 0.01, *** *p* ≤ 0.001, for statistical significance.

The cytotoxicity of the extracts was evaluated in the human HEK293 (served as normal cells), HeLa (cervical cancer), and MCF-7 (breast cancer) cells. The extracts exhibited the highest cytotoxicity at 250 µg/mL (Figure 2) with a dose-dependent effect. The DCM extract again showed the most potent anticancer activity, with IC_50_ values of 273.47 µg/mL (HEK293), 93.82 µg/mL (HeLa), and 144.17 µg/mL (MCF-7) (Table 1). Notably, the extracts exerted greater cytotoxic effects in cancer cells compared to normal cells.

**Figure 3:**
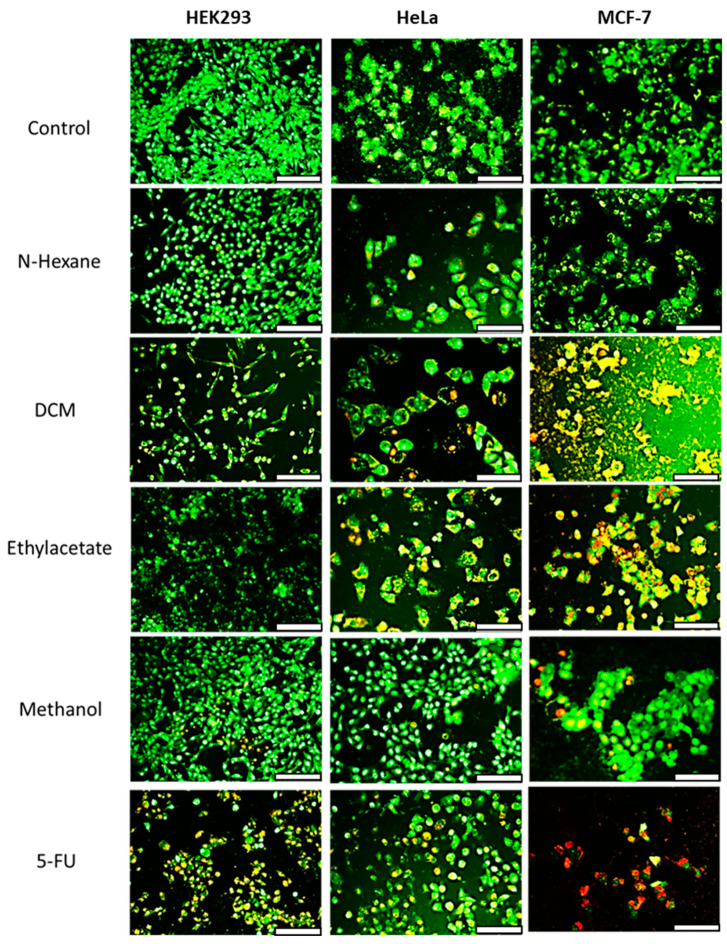
Fluorescent images of acridine orange/ethidium bromide dual-stained HEK293, HeLa and MCF-7 cells treated with 100 µg/mL extracts of *E. caffra*. Scale Bar = 100 µm.

Further insights into the mechanism of cell death were obtained using acridine orange/ethidium bromide (AO/EB) dual staining (Figure 3). Cells treated with the n-hexane extract displayed green fluorescence, indicating viable cells with intact double-stranded DNA. HEK293 cells treated with extracts showed green to orange fluorescence, suggesting early apoptosis. HeLa and MCF-7 cells treated with the extracts exhibited early to late apoptotic features, including condensed green nuclei and red fluorescence indicative of fragmented DNA. The standard anticancer drug 5-FU induced red fluorescence in MCF-7 cells due to ethidium bromide binding to the DNA fragments, confirming apoptosis. Thus, treatment with the extracts induced apoptotic cell death in the cancer cells.

### 3.2. Chemical Characterization of Isolated Compounds

**Figure 4:**
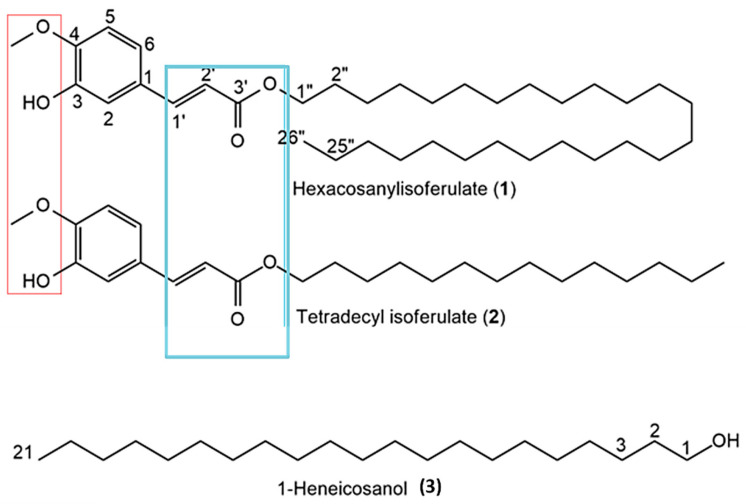
Chemical structures of compounds isolated from *Erythrina caffra*. Red and blue boxes show common functionalities that are likely responsible for bioactivities.

Due to the superior antioxidant and anticancer activity of the DCM extract (Table 1), it was selected for the purification of bioactive compounds via column chromatography. Three different compounds were isolated. All NMR spectra, HR-ESI-MS, and GC–MS data are provided in Supplementary Figure S2a–i. The chemical structures of the isolated compounds, including shared functional groups, are shown in Figure 4.

Compound 1 (201 mg) was isolated as a yellow amorphous solid with molecular formula C_36_H_62_O_4_, confirmed by HR-ESI-MS (*m*/*z* 557.4571 [M–H]^−^; calcd 557.4570). Its ^1^H NMR (400 MHz, CDCl_3_) data: δ_H 0.85 (3H, t, J = 6.5 Hz; terminal CH_3_), 1.19–1.31 (–(CH_2_)_n_–), 3.90 (3H, s; 4-OMe), 4.16 (2H, t, J = 6.7 Hz; –OCH_2_–), 6.28 (1H, d, J = 15.9 Hz; =CH, H-2’), 6.89 (1H, d, J = 8.1 Hz; H-5), 7.01 (H-2), 7.06 (H-6), 7.60 (1H, d, J = 15.9 Hz; =CH, H-’). ^13^C NMR (CDCl_3_): δ_C 14.1, 22.7, 25.8, 28.8, 29.3–29.6, 31.9, 55.9 (4-OMe), 64.7, 109.3, 114.7, 115.6, 123.0, 125.8, 144.7, 168.2. Based on a literature comparison, compound 1 was identified as n-Hexacosanyl isoferulate [17].

Compound 2 (137 mg) was a yellow solid with molecular formula C_24_H_38_O_4_ from HR-ESI-MS (*m*/*z* 391.2844 [M+H]^+^; calcd 391.2848). Its ^1^H NMR data closely resembled compound 1, but with differences indicating a shorter hydrocarbon chain (δ_H 0.85 (3H, t, J = 6.6 Hz), 1.17–1.37 (–(CH_2_)_n_–), 3.90 (3H, s; 4-OMe), 4.16 (2H, t, J = 6.7 Hz), 6.28 (1H, d, J = 15.9 Hz), 6.90 (1H, d, J = 8.1 Hz), 7.01 (1H, d, J = 1.6 Hz), 7.06 (1H, dd, J = 8.1 and 1.6 Hz), 7.60 (1H, d, J = 15.9 Hz)). The compound was characterized as tetradecyl isoferulate, a derivative similar to Tetradecyl ferulate previously found in *Erythrina sigmoidea* [17].

Compound 3 (73 mg) was a white crystalline solid identified as 1-heneicosanol with molecular formula C_21_H_44_O (mass 312, GC–MS data). Its ^1^H NMR showed typical fatty alcohol signals: δ_H 0.85 (3H, t, J = 6.8 Hz; H-21), 1.23–1.56 ((CH_2_)_19_; H-2–H-20), 3.61 (2H, t, J = 6.6 Hz; H-1), and ^13^C NMR signals at δ_C 14.1, 22.7, 25.7, 29.3–29.7, 31.9, 32.8, 63.1, consistent with the literature for 1-Heneicosanol from *Senecio coluhuapiensis* [18].

### 3.3. Anticancer Activity of Isolated Bioactive Compounds from Erythrina caffra

The isolated compounds were assessed for anticancer activity against normal and cancer cell lines. All fractions showed significant dose-dependent cytotoxic effects (Table 2).

**Figure 5:**
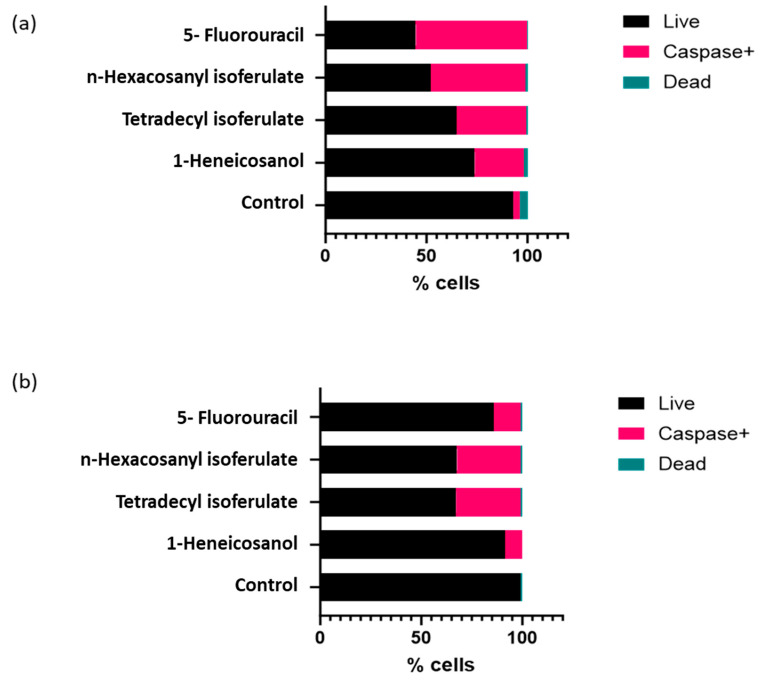
Image showing the effects of the identified *E. caffra* fractions on caspase expression in the (**a**) HeLa and (**b**) MCF-7 cells.

**Figure 6:**
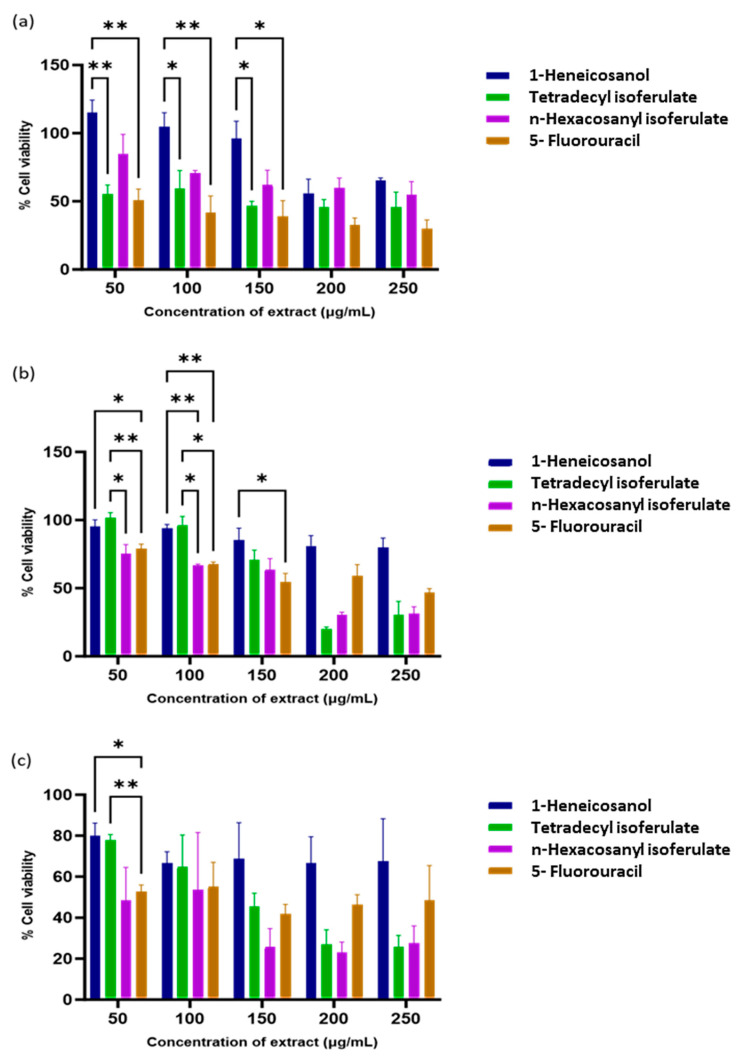
Cytotoxicity of *E. caffra* fractions on: (**a**) HEK293, (**b**) HeLa, and (**c**) MCF-7 cells. Data are presented as mean percentage cell viability ± SD (*n* = 3). * *p* < 0.05 and ** *p* ≤ 0.01 for statistical significance.

From the isolated compounds, 1-heneicosanol exhibited the weakest cytotoxicity, while the ferulate derivatives—n-hexacosanyl isoferulate and tetradecyl isoferulate—demonstrated comparable and significant anticancer effects. Notably, n-hexacosanyl isoferulate demonstrated the most potent anticancer activity, with IC_50_ values of 58.8 µg/mL and 146.6 µg/mL in the MCF-7 and HeLa cells (Table 2). The ferulic acid derivatives, tetradecyl isoferulate and n-hexacosanyl isoferulate, demonstrated pronounced caspase activation potential (Figure 5 and Figure 6, Supplementary Figure S3).

## 4. Discussion

This study presents significant evidence that the stem bark of Erythrina caffra contains bioactive compounds with antioxidant and anticancer properties. These findings extend previous investigations on other Erythrina species and support the growing body of evidence suggesting that this genus possesses considerable antioxidant and anticancer potential [9,19,20].

The DCM extract of *E. caffra* showed the highest antioxidant activity from all tested fractions, with an IC_50_ of 144.17 µg/mL. While antioxidant and anticancer activities have previously been reported in other *Erythrina* species such as *E. abyssinica* and *E. variegata*, the current study is the first to our knowledge to specifically examine the stem bark of *E. caffra* [21,22]. The observed antioxidant effects are likely due to phenolic compounds, especially ferulic acid derivatives, which are known for their ability to neutralize reactive oxygen species and protect biomolecules from oxidative damage [23]. The hydrogen of the hydroxyl group is known to react with free radicals. The antioxidant activity further depends on the groups of the carbon side chain and the positioning of the methoxyl and hydroxyl groups on the aromatic ring [24]. Free radicals are reported to play a crucial role in cancer development. Since ferulic acid and its derivatives can scavenge ROS, enhance the activity of cytoprotective enzymes such as UDP-glucuronosyl transferases in the liver, and detoxification of carcinogenic compounds [25], they have the potential to act as anticancer agents. Since oxidative stress plays a major role in cancer development, these findings suggest that *E. caffra* could have significant potential in cancer prevention.

In addition to the favorable antioxidant effects, the DCM extract of *E. caffra* exhibited selective cytotoxicity in the HeLa and MCF-7 cancer cells, while exerting significantly lower toxicity on the non-cancer HEK293 cells. This selective effect is a desirable property in the development of anticancer agents, as many conventional chemotherapeutics lack specificity and damage both malignant and healthy cells, leading to adverse side effects [26]. A comparable pattern of selective toxicity has been reported for compounds isolated from *Erythrina excelsa* and *Erythrina senegalensis* on sensitive and drug-resistant cancer cell lines [27]. Apoptotic cell death due to plant extracts has been reported previously using acridine orange/ethidium bromide (AO/EB) staining to identify classic apoptotic features, such as red-orange fluorescence, chromatin condensation and membrane blebbing, while viable cells emitted a green fluorescence [28]. These results are favorable compared to those reported using the anticancer drug doxorubicin alone and complexed to bimetallic [29] and magnetic [30] nanoparticles. Furthermore, these observations also align with known bioactivities of *Erythrina*-derived compounds, including alkaloids and flavonoids. It was reported that 4′-methoxy licoflavanone and alpinumisoflavone, flavonoids isolated from *Erythrina suberosa,* can induce apoptosis in leukemia HL-60 cells by disrupting mitochondrial membrane potential and activating the caspase cascade [19].

A significant outcome of this study was the isolation of the three key compounds (n-hexacosanyl isoferulate, tetradecyl isoferulate, and 1-heneicosanol) from the DCM extract, which showed significant antioxidant and anticancer effects. Among them, the isoferulate derivatives showed more potent anticancer activity. Ferulic acid has been previously reported to exhibit anticancer effects, which are mediated through modulation of key signalling pathways, including PI3K/Akt and MAPK, and the induction of p53-dependent apoptosis [31]. Interestingly, the n-hexacosanyl isoferulate was more cytotoxic than tetradecyl isoferulate, possibly due to its longer aliphatic chain, which might enhance its ability to penetrate cell membranes [32].

Of note was the activation of caspases by the isoferulate compounds, which are also shown to be good antioxidants. This suggests that apoptosis is triggered by activating the caspase cascade, thereby enhancing its cytotoxic efficacy against cancer cells. Similar caspase-driven apoptosis has been reported in studies on ferulic acid, highlighting its potential as a multitargeted cancer therapy. Overall, this research underscores the promising role of plant-derived phenolics in cancer treatment. Compared to synthetic compounds, natural isoferulates like those found in *E. caffra* offer a safer and potentially more effective option for developing new cancer therapeutics.

## 5. Conclusions

The results have thus far shown that the *E. caffra* bark extract contains bioactive constituents such as 1-heneicosanol, tetradecyl isoferrulate and n-hexacosanyl isoferrulate, with significant biomedical potential. Of these, n-hexacosanyl isoferrulate was found to have the most potent anticancer effects, with the ability to induce apoptosis and cell death in cervical cancer (HeLa) and breast cancer (MCF-7) cells by triggering the caspase protein cascade. Hence, this lead compound warrants further investigation in more cell lines and in an in vivo model. Further mechanistic studies could also unravel more information on its mechanism of action. This could entail investigating specific signalling pathways, such as the PI3K/Akt and MAPK pathways, and examining the expression or regulation of important pro-apoptotic and anti-apoptotic genes. Based on the promising in vitro results obtained, selected cancer mouse models can be utilized for in vivo studies to identify cancer type specificity.

## Figures and Tables

**Table 1:** IC_50_ values (µg/mL) of *E. caffra* bark extract in DPPH radical scavenging and anticancer activities.

Extracts/Standard/Drug	DPPH (µg/mL)	HEK293 (µg/mL)	MCF-7 (µg/mL)	HeLa (µg/mL)
Hexane	84,990.83	996.55	4213.47	84,990.83
DCM	144.17	273.47	93.82	144.17
Ethylacetate	356.39	138.16	795.06	356.39
Methanol	208.18	108.45	295.61	208.18
Ascorbic acid	84.37	-	-	-
5-FU	-	54.50	147.90	510.26

**Table 2:** The IC_50_ values of isolated compounds from *Erythrina caffra* in the HEK293, MCF-7, and HeLa cells.

Extracts/Standard	HEK293 (µg/mL)	MCF-7 (µg/mL)	HeLa (µg/mL)
1-Heneicosanol	371.5874	1979.46	4013.47
Tetradecyl isoferulate	140.6733	123.6174	169.80
n-Hexacosanyl isoferulate	316.1851	58.83905	146.63

## Data Availability

The data and contributions presented in the study are included in the article and Supplementary File. Further inquiries can be directed to the corresponding author.

## Supplementary Materials

The following supporting information can be downloaded at: https://www.mdpi.com/article/10.3390/antiox14091035/s1. Supplementary Figure S1: Flow chart for the purification of anticancer compounds from *Erythrina caffra*; Supplementary Figure S2: ^1^H and ^13^C NMR spectrum of compounds 1–3; Supplementary Figure S3: Quantitative analysis of multicaspase expression in HeLa and MCF7 cells using flow cytometry.

## Abbreviations

The following abbreviations are used in this manuscript:

DPPH	2,2-diphenyl-1-picrylhydrazyl
MTT	3-(4,5-dimethylthiazol-2-yl)-2,5-diphenyltetrazolium bromide
DCM	Dichloromethane
MeOH	Methanol
HEK293	Human Embryonic Kidney 293 cells
HeLa	Human cervical cancer cell line
MCF-7	Human breast cancer cell line
IC_50_	Half-maximal inhibitory concentration
AO/EB	Acridine Orange/Ethidium Bromide
5FU	5-Fluorouracil
CC	Column Chromatography
TLC	Thin-Layer Chromatography
NMR	Nuclear Magnetic Resonance
^1^H NMR	Proton Nuclear Magnetic Resonance
^13^C NMR	Carbon-13 Nuclear Magnetic Resonance
HRMS	High-Resolution Mass Spectrometry
HR-ESI-MS	High-Resolution Electrospray Ionization Mass Spectrometry
GC–MS	Gas Chromatography–Mass Spectrometry
CDCl_3_	Deuterated Chloroform
PBS	Phosphate-Buffered Saline
DMSO	Dimethyl Sulfoxide
NAD(P)H	Nicotinamide Adenine Dinucleotide (Phosphate)
7-AAD	7-Aminoactinomycin D
MAPK	Mitogen-Activated Protein Kinase
PI3K/Akt	Phosphoinositide 3-Kinase/Protein Kinase B
