# Supplementary material for: Isolation, Characterization, Antioxidant and Anticancer Activities of Compounds from Erythrina caffra Stem Bark Extract
Source: Antioxidants (Basel). 2025 Aug 22;14(9):1035. doi: 10.3390/antiox14091035 (PMC12466456; doi:10.3390/antiox14091035)
Supplement: Supplementary file 1 [file antioxidants-14-01035-s001.zip › antioxidants-3798321-supplementary.pdf]

antioxidants

Supplementary Material

Isolation, Characterization, Antioxidant and Anticancer Activities of Compounds from *Erythrina caffra* Stem Bark Extract

Femi Olawale [1], Olusola Bodede [2], Mario Ariatti [1] and Moganavelli Singh [1,*]

[1] Nano-Gene and Drug Delivery Group, Discipline of Biochemistry, University of KwaZulu-Natal, Private Bag X54001, Durban 4000, South Africa; olawalefemi3@gmail.com (F.O.); ariattim@ukzn.ac.za (M.A.)
[2] Biodiscovery Centre, Department of Chemistry, University of Pretoria, Pretoria 0028, South Africa; olusolabodede@northeastern.edu
* Correspondence: singhm1@ukzn.ac.za; Tel.: +27-31-2607170

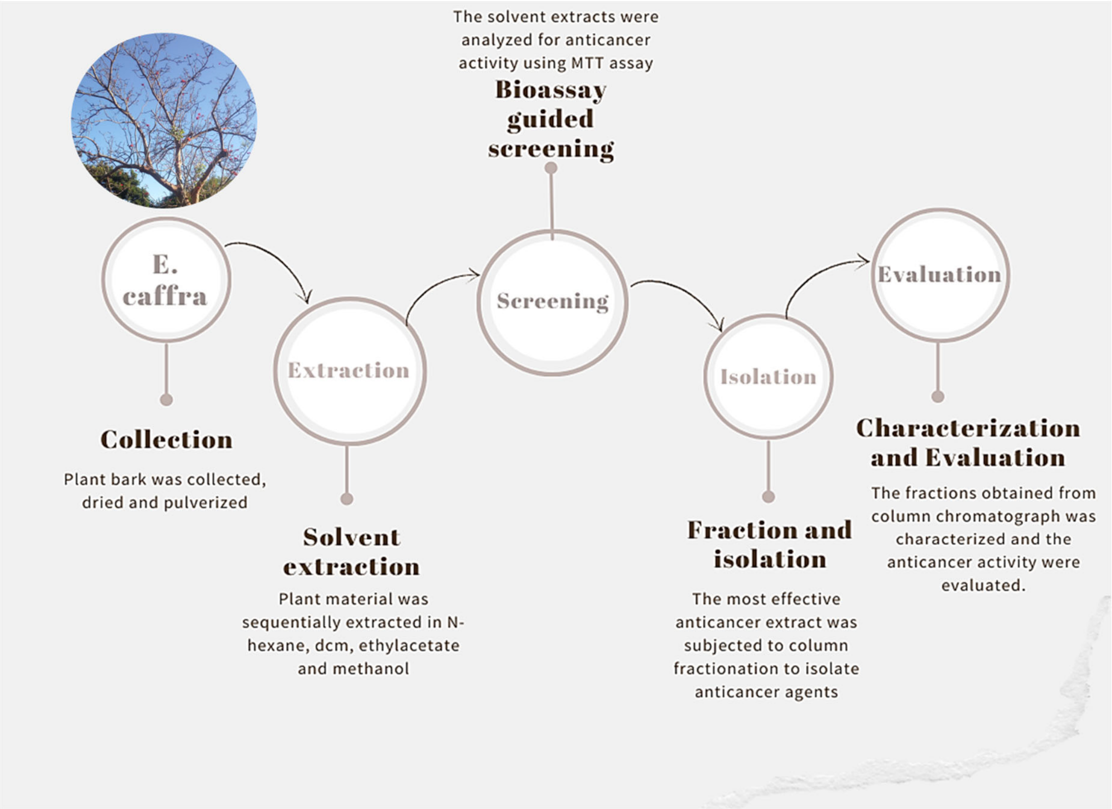

Supplementary Figure S1. Flow chart for the purification of anticancer compounds from *Erythrina caffra*.

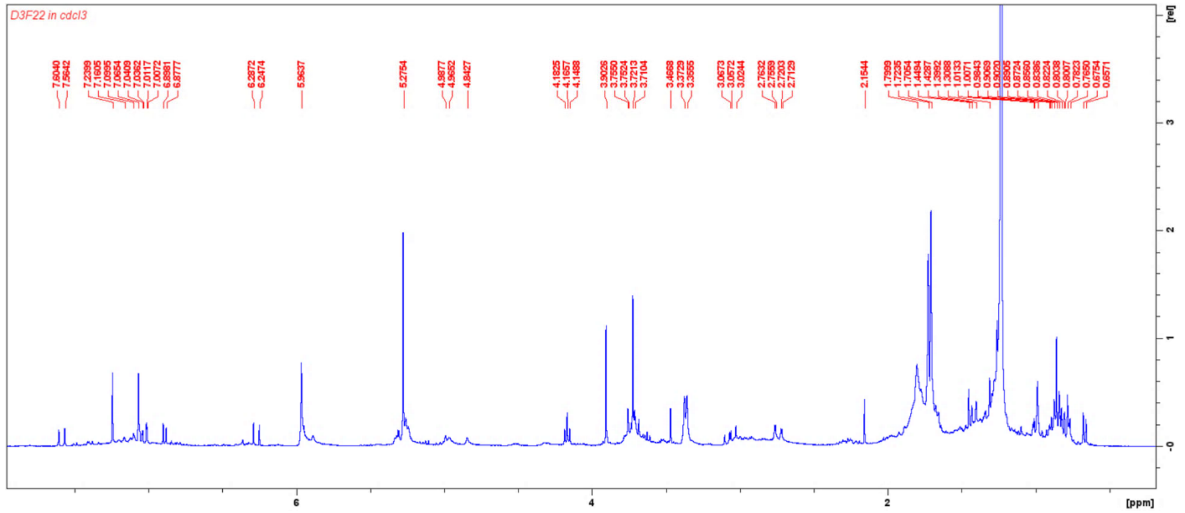

Supplementary Figure S2a. ^{1}H NMR spectrum of compound 1.

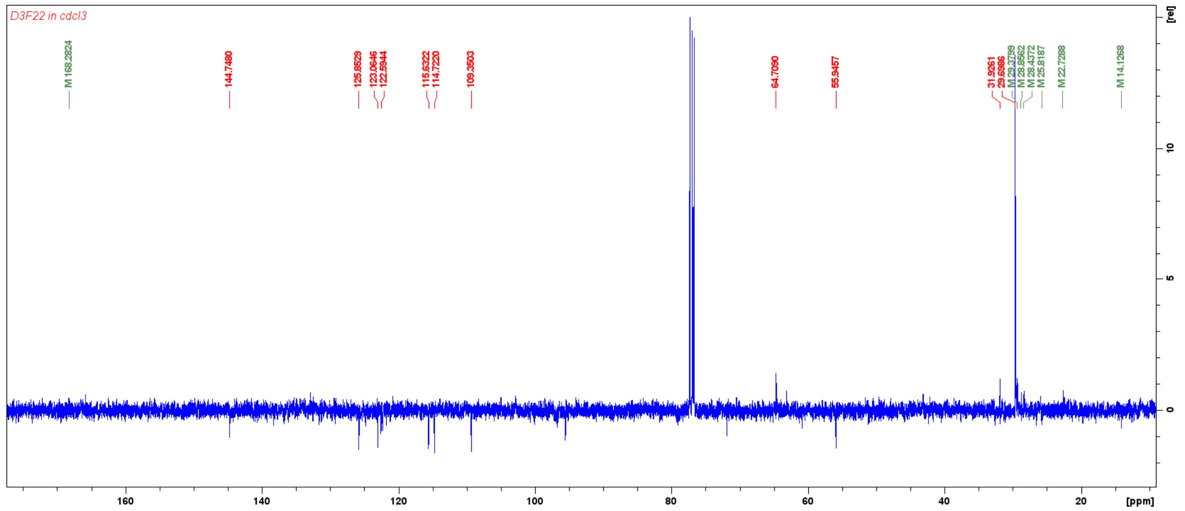

Supplementary Figure S2b. ^{13}C NMR spectrum of compound 1.

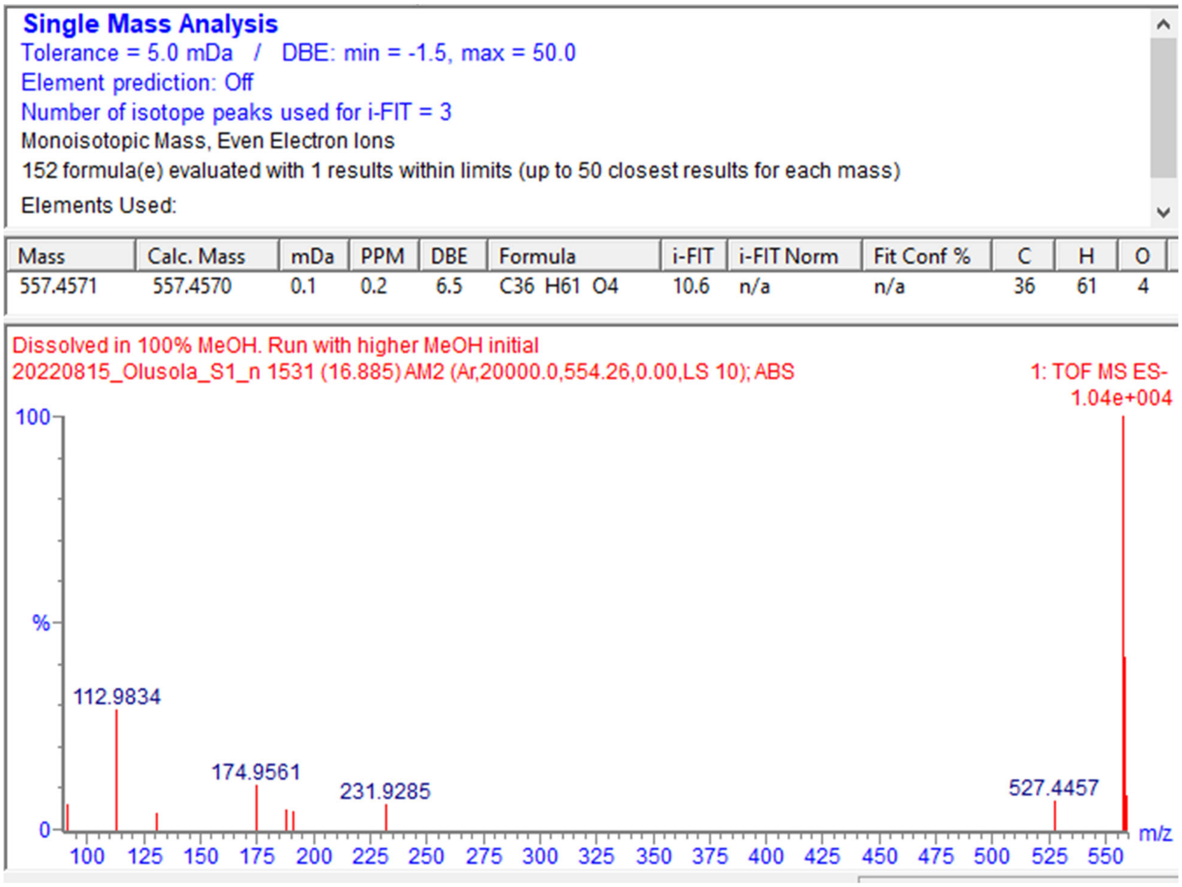

Mass	Calc. Mass	mDa	PPM	DBE	Formula	i-FIT	i-FIT Norm	Fit Conf %	C	H	O
557.4571	557.4570	0.1	0.2	6.5	C36 H61 O4	10.6	n/a	n/a	36	61	4

Supplementary Figure S2c. HR-ESI-MS spectrum of compound 1.

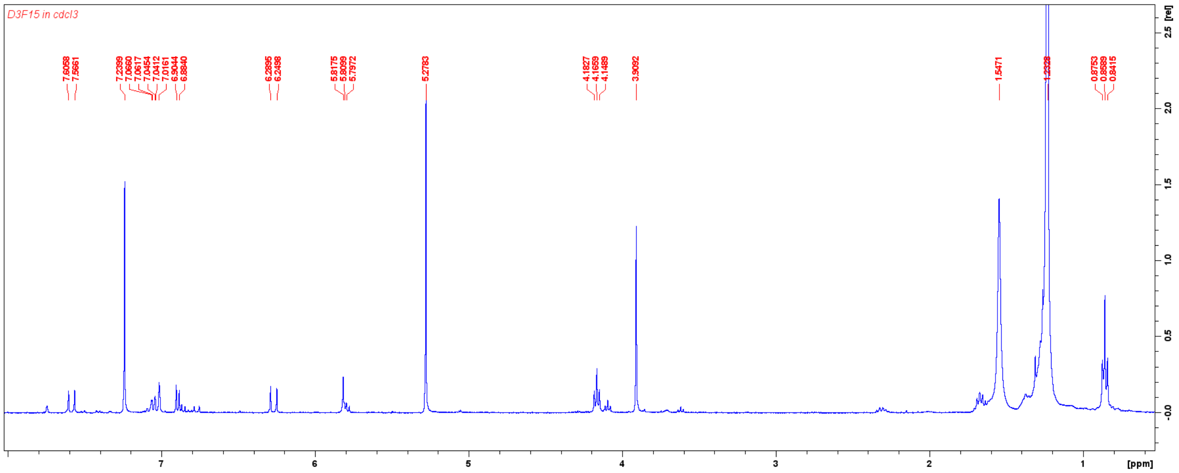

Supplementary Figure S2d. ^{1}H NMR spectrum of compound 2

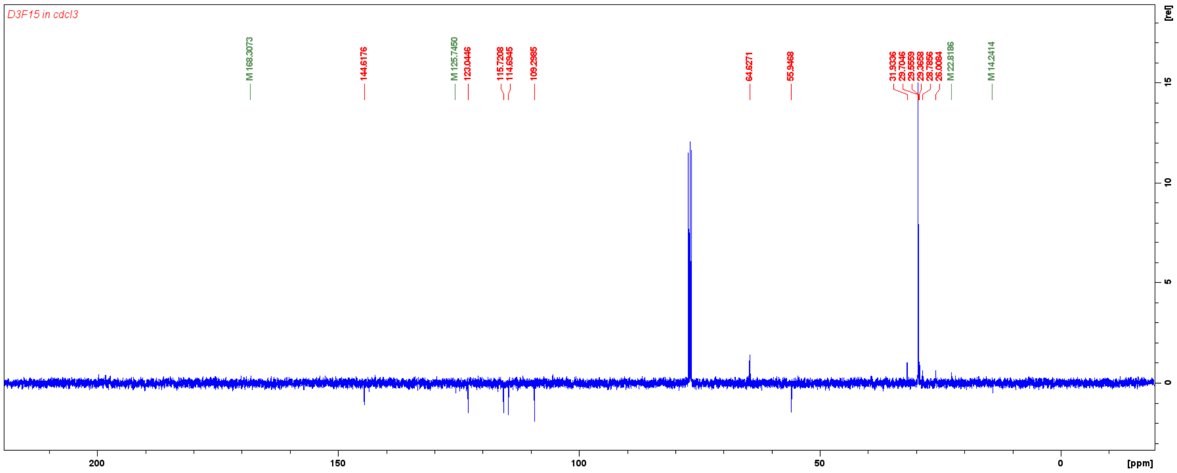

Supplementary Figure S2e. ^{13}C NMR spectrum of compound 2

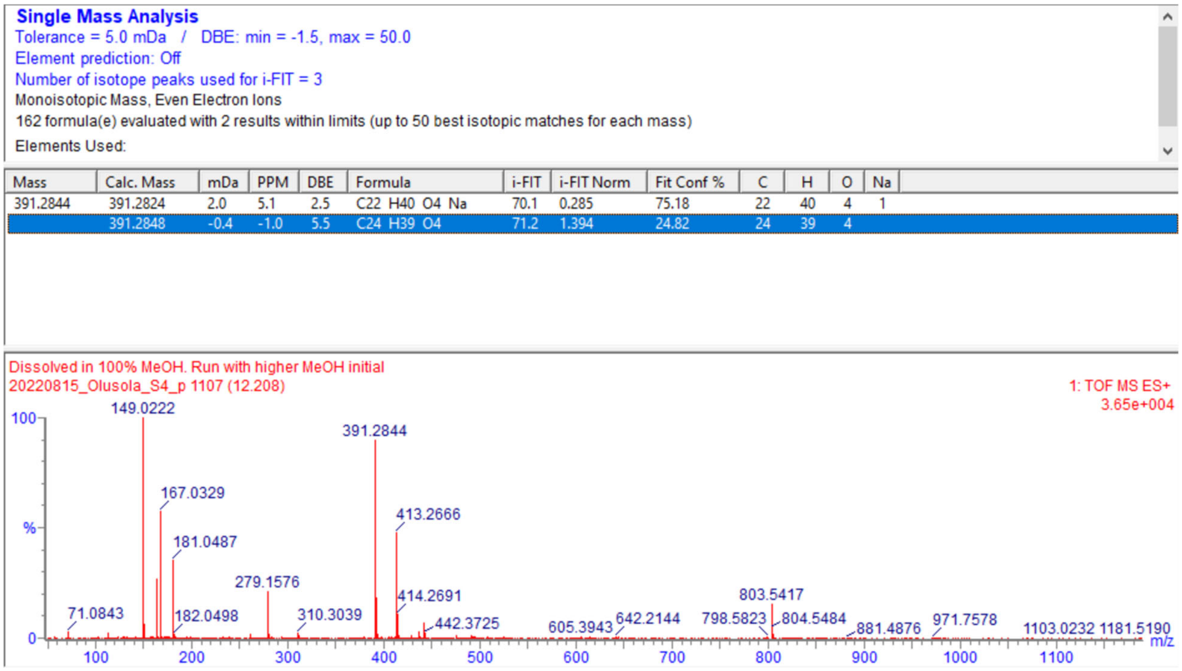

Mass	Calc. Mass	mDa	PPM	DBE	Formula	i-FIT	i-FIT Norm	Fit Conf %	C	H	O	Na
391.2844	391.2824	2.0	5.1	2.5	C22 H40 O4 Na	70.1	0.285	75.18	22	40	4	1
	391.2848	-0.4	-1.0	5.5	C24 H39 O4	71.2	1.394	24.82	24	39	4	

Supplementary Figure S2f. HR-ESI-MS spectrum of compound 2

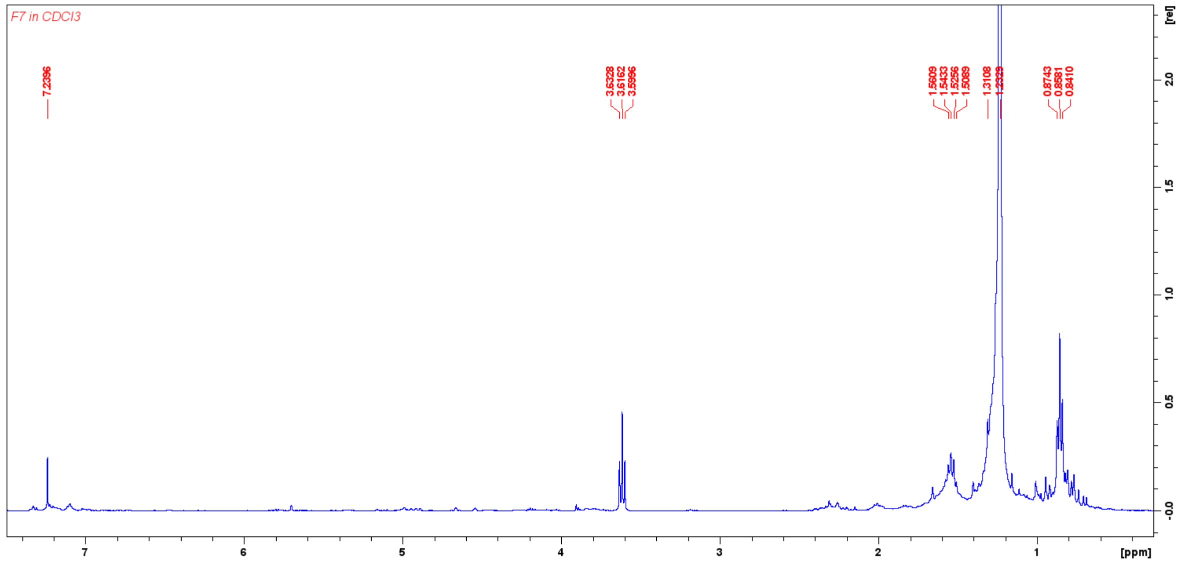

Supplementary Figure S2g. ^{1}H NMR spectrum of compound 3

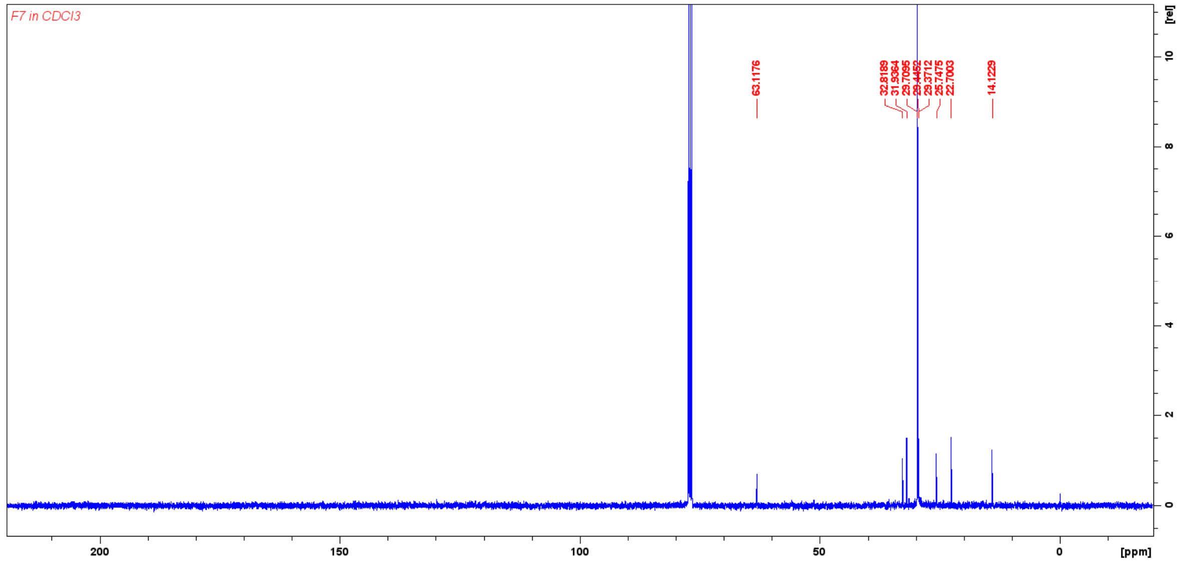

Supplementary Figure S2h. ^{13}C NMR spectrum of compound 3

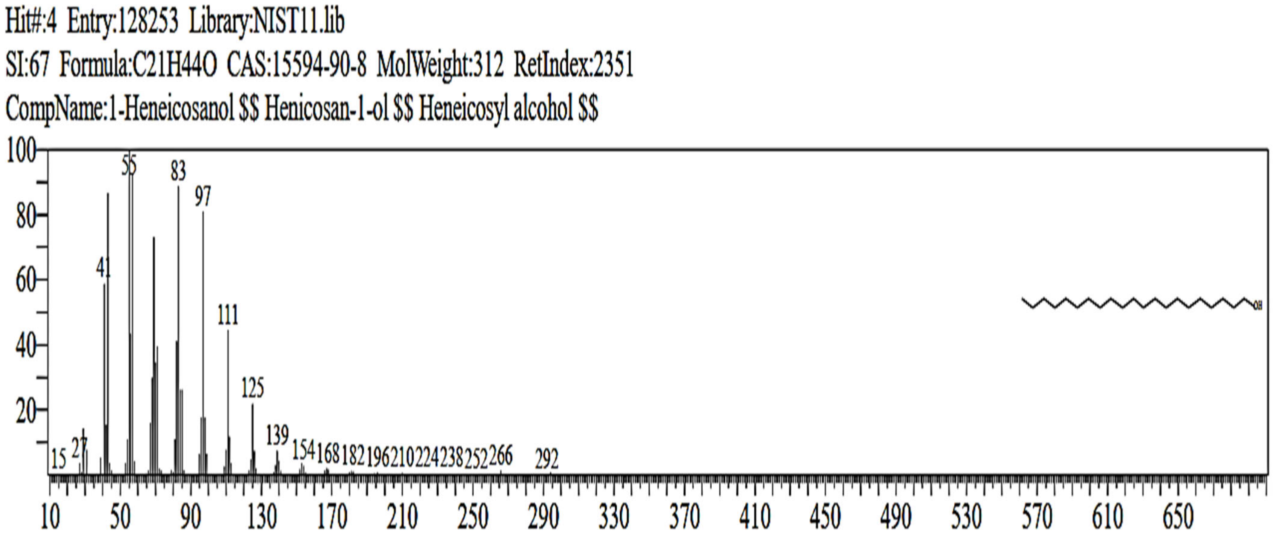

Supplementary Figure S2i. GC-MS spectrum of compound 3

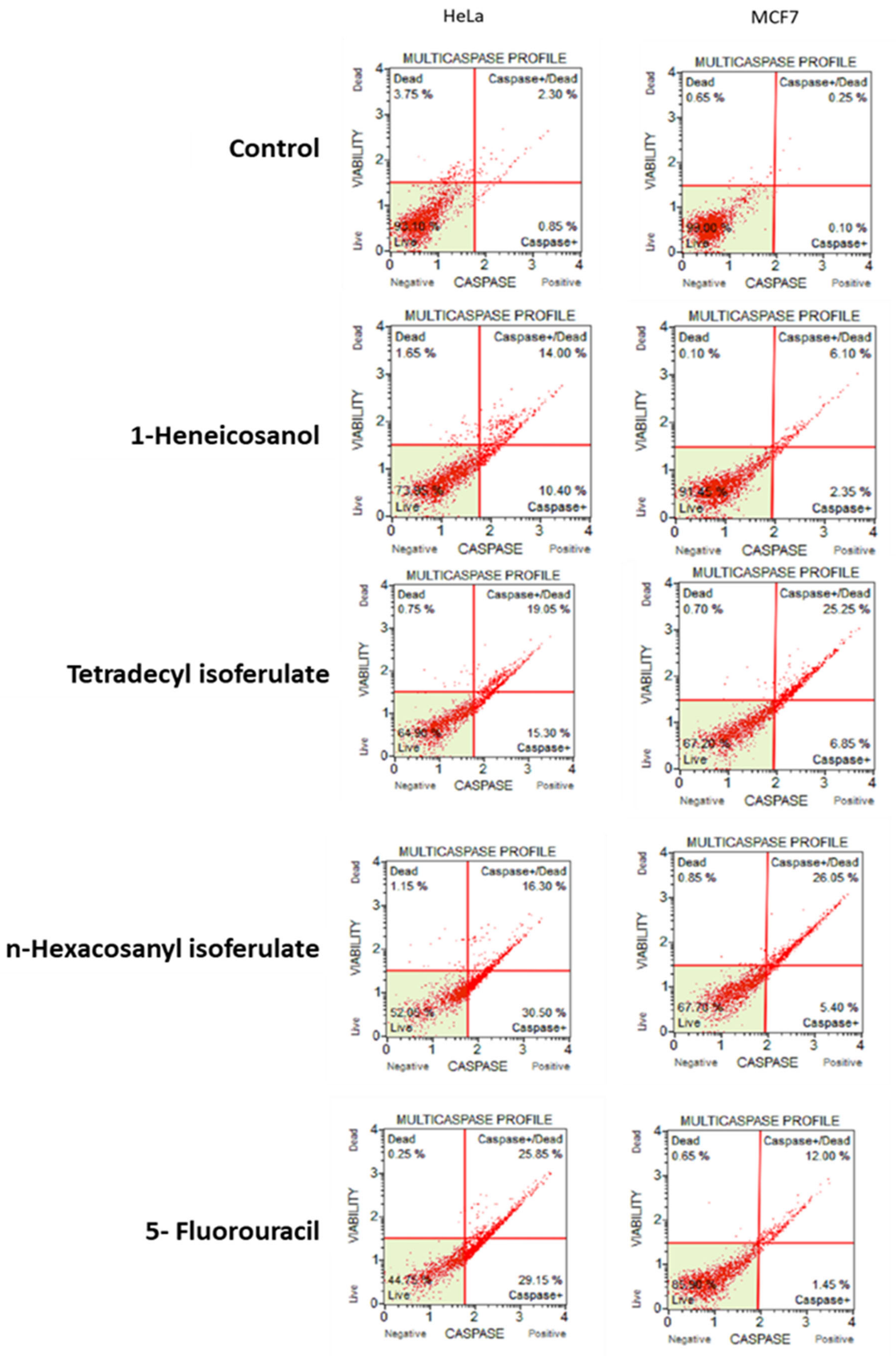

Supplementary Figure S3. Quantitative analysis of multicaspase expression in HeLa and MCF-7 cells using flow cytometry.
